# Microbiota in Pancreatic Diseases: A Review of the Literature

**DOI:** 10.3390/jcm10245920

**Published:** 2021-12-17

**Authors:** Tommaso Schepis, Sara S. De Lucia, Enrico C. Nista, Vittoria Manilla, Giulia Pignataro, Veronica Ojetti, Andrea Piccioni, Antonio Gasbarrini, Francesco Franceschi, Marcello Candelli

**Affiliations:** 1 Medical and Surgical Science Department, Fondazione Policlinico Universitario Agostino Gemelli—IRCCS, Università Cattolica del Sacro Cuore di Roma, 00168 Roma, Italy; tommaso.schepis@gmail.com (T.S.); sarasdelucia@gmail.com (S.S.D.L.); enricocelestino.nista@policlinicogemelli.it (E.C.N.); vittoriamanilla@hotmail.it (V.M.); antonio.gasbarrini@policlinicogemelli.it (A.G.); 2 Emergency Medicine Department, Fondazione Policlinico Universitario Agostino Gemelli—IRCCS, Università Cattolica del Sacro Cuore di Roma, 00168 Roma, Italy; giulia.pignataro@policlinicogemelli.it (G.P.); veronica.ojetti@policlinicogemelli.it (V.O.); andrea.piccioni@policlinicogemelli.it (A.P.); francesco.franceschi@policlinicogemelli.it (F.F.)

**Keywords:** microbiota, acute pancreatitis, pancreatic cancer, chronic pancreatitis, autoimmune pancreatitis

## Abstract

The gut microbiota is a critical element in the balance between human health and disease. Its impairment, defined as dysbiosis, is associated with gastroenterological and systemic diseases. Pancreatic secretions are involved in the composition and changes of the gut microbiota, and the gut microbiota may colonize the pancreatic parenchyma and be associated with the occurrence of diseases. The gut microbiota and the pancreas influence each other, resulting in a “gut microbiota-pancreas axis”. Moreover, the gut microbiota may be involved in pancreatic diseases, both through direct bacterial colonization and an indirect effect of small molecules and toxins derived from dysbiosis. Pancreatic diseases such as acute pancreatitis, chronic pancreatitis, autoimmune pancreatitis, and pancreatic cancer are common gastroenterological diseases associated with high morbidity and mortality. The involvement of the microbiota in pancreatic diseases is increasingly recognized. Therefore, modifying the intestinal bacterial flora could have important therapeutic implications on these pathologies. The aim of this study is to review the literature to evaluate the alterations of the gut microbiota in pancreatic diseases, and the role of the microbiota in the treatment of these diseases.

## 1. Introduction

The human gut is colonized by 100 trillion microorganisms and over 1000 different resident bacterial species [1]. The entire microbiota inhabiting the gut is referred to as the “gut microbiota”, while their genomes are collectively known as the “gut microbiome” [1]. The gut microbiota is composed of microbes from different kingdoms, including bacteria, fungi, and viruses [2]. The gut microbiota is emerging as a critical determinant of human body homeostasis and the balance between health and disease. An imbalance in the gut microbiota is termed “dysbiosis” and is associated not only with gastrointestinal diseases (e.g., celiac disease, inflammatory bowel disease, irritable bowel syndrome, colonic diverticulosis, non-alcoholic fatty liver disease), but also with systemic diseases such as obesity, Alzheimer’s disease, metabolic syndrome, and diabetes mellitus [3,4,5,6,7]. The gut microbiota is highly individualized and easily altered by internal and external factors [8]. Genetics, age, comorbidities, medications, hygiene, socioeconomic status, diet, occupation, and the immune system are some of the determinants of changes in the gut microbiota [2]. Older age is also associated with changes in the gut microbiota, probably as a result of the immune phenomena associated with aging (immunosenescence) [9]. Several studies support the existence of a gut microbiota–pancreas axis that influences the other. On the one hand, pancreatic secretions may alter the gut microenvironment and play a role in the composition of the gut microbiota, and on the other hand, the gut microbiota may influence the occurrence of pancreatic diseases [10]. The pancreas is anatomically directly connected to the gastrointestinal tract through the pancreatic duct system, which leads to an inevitable connection with the gut microbiota. It was once believed that the pancreas was a sterile organ, but several studies have shown that physiologically, the pancreas itself has a microbiota [11]. The pathways by which microbes can enter the pancreas are controversial, and several hypotheses have been proposed. The simplest theory involves the translocation of duodenal microbiota into pancreatic tissue via the pancreatic ductal system [12]. The translocation of gut microbiota may also occur via the mesenteric venous drainage or the mesenteric lymphatic system [13]. Not only can the gut microbiota colonize and influence pancreatic health, but the physiological activities of the pancreas (e.g., pancreatic juice production) can also alter the composition of the gut microbiota. Pancreatic enzymes are responsible for the digestion of food, and their absence may cause undigested food to remain in the colon and promote bacterial overgrowth [14]. In addition, the exocrine pancreas also appears to produce antimicrobial peptides that may alter microbiota assessment [15]. Pietzner M. et al. collected 2226 stool and blood samples to determine the pancreatic exocrine function (pancreatic elastase enzyme), intestinal microbiota profiles, and plasma metabolic levels [14]. They reported that pancreatic exocrine function was simultaneously associated with changes in intestinal microbiota and plasma metabolites. Their results suggest that pancreatic secretions influence the composition of the gut microbiota and alter the availability of metabolites derived from the microbiome, resulting in systemic resonance. The link between the gut microbiota and the occurrence of the pancreatic disease is increasingly recognized. The gut barrier failure that occurs in acute pancreatitis allows the translocation of gut microbes and endotoxins that contribute to the local and systemic severity of acute pancreatitis [16]. In addition, the bacterial overgrowth of the small intestine is a common finding in chronic pancreatitis and is reported to be an important factor in symptoms, malnutrition, and morbidity [17]. The role of microbiota has also been described in the pathogenesis, progression, and response to therapy of pancreatic cancer [18]. The relationship between pancreatic disease and the gut microbiota is determined by the interplay of the immune system, proinflammatory status, and dysbiosis [10]. In this article, we review the current literature to evaluate the role of gut microbiota in pancreatic diseases such as acute pancreatitis, chronic pancreatitis, autoimmune pancreatitis, pancreatic cancer, and NAFPD.

## 2. Materials and Methods

Articles were identified using PubMed, Scopus, and Embase databases by searching with the following keywords: “microbiota”, “gut microbiota”, “probiotics”, “prebiotics”, “microbiota fecal transplant”, “MFT”, “pancreas”, “acute pancreatitis”, “chronic pancreatitis”, “pancreatic cancer”, “non-alcoholic fatty pancreatic disease”, “NAFPD” and “autoimmune pancreatitis” Only articles published after 1990 were considered. The authors individually reviewed English-language articles for relevance, and the results were compared to include only the most relevant articles. Letters, commentary, and opinions were not included in the search.

## 3. Acute Pancreatitis

Acute pancreatitis (AP) is an inflammatory disease characterized by pain in the upper abdomen and the elevation of pancreatic enzymes. The incidence of AP is increasing year by year, and it is now one of the most common gastrointestinal disorders requiring acute hospitalization worldwide [19]. Gallstones and chronic alcohol consumption account for about two-thirds of cases. However, the incidence of hypertriglyceridemia-associated acute pancreatitis (HTGAP) continues to increase, contributed by rapid economic growth and worldwide changes in dietary patterns [20]. Acute pancreatitis continues to be associated with high mortality today, with two peaks of mortality, early and late (the “second hit”), defined as less or more than one week after admission, respectively [21]. The relationship between AP and gut microbiota has been widely studied. In 1996, C. D. Johnson et al. first demonstrated the importance of early antibiotic prophylaxis in AP reducing systemic gastrointestinal bacterial shifts and postulated the value of gut decontamination by oral antibiotics for the treatment of AP [22]. Many studies have shown that gut dysfunction plays a leading role in the development of severe forms of AP since the role of the gut microbiota in inflammatory and chronic diseases was discovered. Alterations in microcirculation due to the massive release of inflammatory cytokines and hypovolemia lead to ischemia of the intestinal mucosa, which in combination with reperfusion injury (often due to fluid resuscitation) leads to the loss of intestinal barrier function and the subsequent vascular translocation of gastrointestinal bacteria. These findings prompted researchers to question whether differences in individual microbiome patterns and/or dysbiosis might influence the prognosis of AP. Chaochao T. et al. conducted a multicenter prospective study involving 108 subjects (44 with severe AP, 32 with mild AP, and 32 healthy controls), and collected stool and blood samples for the detection of intestinal bacteria and endotoxins, respectively [23]. Dramatic changes in the richness and diversity of the gut microbial pattern were found in patients with AP, with large differences between severe and mild AP. In addition, rates of multiorgan failure and infectious complications were higher in patients with severe AP with altered gut microbiota than in patients with unaltered gut microbiota. In severe AP, an increase in Enterococcus and a decrease in Bifidobacterium have been documented. In addition, a positive association between Enterobacteriaceae and systemic inflammation (serum levels IL-6) was described. In a case-control study, the gut microbiota of 45 patients with acute pancreatitis and 44 healthy volunteers was compared. A total of 27 microbial phyla were detected, with samples from patients with AP containing fewer phyla. Compared to healthy individuals, increased levels of Bacteroidetes and Proteobacteria and lower levels of Firmicutes and Actinobacteria were detected in AP patients [24]. Similarly, Shanshan Y. et al. studied 80 patients (20 with mild AP, 20 with mild-severe AP, 20 with severe AP, and 20 healthy subjects) and documented a significantly high incidence of dysbiosis in patients with AP, with differences in the composition of the gut microbiota of the three different severity grades. Bacteroides, Escherichia-Shigella, and Enterococcus were respectively the predominant gut species in mild AP, mild-severe AP, and severe AP [25]. Interestingly, an increased abundance of Escherichia-Shigella was found not only in rectal swabs, but also in the peripheral blood of patients with AP [26]. Escherichia-Shigella can disrupt intestinal mucosal barriers and contribute to severe local and systemic inflammation. On the other hand, several studies show increased levels of Blautia and Bifidobacterium in the gut microbiota of healthy volunteers compared to patients with AP. Blautia can produce short-chain fatty acids (SCFAs), important sources of energy for the intestinal mucosa, which play a protective role in intestinal homeostasis [27]. Xiaomin H. et al. published a study aiming to explore the differences between hypertriglyceridemia-induced pancreatitis (HTGAP) and non-HTGAP. They also investigated the relationship between the gut microbiome and prognosis in patients with HTGAP [20]. HTGAP was associated with a more severe prognosis than non-HTGAP, and an analysis of the gut microbiome revealed a lower abundance of Lachnospiraceae and Bacteroidaceae in the HTGAP group compared with the non-HTGAP group.

Although the role that changes in the gut microbiota play in AP has been widely described, its therapeutic role is still debated. Jakub W. R. et al. showed that probiotics can reverse the gut barrier failure in the late phase of AP, but only if administered before the onset of AP [28]. Similarly, Shuhei T. et al. documented in an animal model that the oral administration of Lactobacillus brevis-derived polyphosphate for 24 days before the induction of AP by caerulein, attenuated inflammation in mice [29]. K. Minaga et al. attributed the preventive effect of polyphosphate on AP to the suppression of pancreatic chemokine production, particularly C-C motif chemokine ligand 2 (CCL2), but also to the increased expression of the tight junction (TJ) proteins zonula occludens (ZO)-1 and occludin in the colon, a part of the intestinal TJ that regulates paracellular permeability [30]. Zao, Han-bing et al. investigated the effect of Clostridium butyricum and its metabolite butyrate in induced SAP with abdominal hypertension (IAH) in mice. Although positive results were obtained in preclinical studies, the use of probiotics in clinical practice in AP patients remains unclear. In 2008, the highly acclaimed PROPATRIA (Probiotic Pancreatitis Trial) study showed no beneficial effect of probiotic prophylaxis on the development of infectious complications [31]. Moreover, mortality was higher in the treated group than in the placebo group. In 2014, Mark C. van Baal et al. reanalyzed the results of the PROPATRIA study in rats, and found that mortality was not increased in the probiotic prophylaxis-treated group [32]. In 2016, a re-evaluation of PROPATRIA suggests that probiotic therapy resulted in higher mortality, due to increased lactic acid produced by the bacterial fermentation of carbohydrates. They therefore conclude that probiotic therapy can be started immediately after the first appearance of disease symptoms in conjunction with a reduced intake of fermentable carbohydrates to prevent bacterial overgrowth [33]. Tian et al. again discussed the results of PROPATRIA and postulated that the duration of treatment and the bacterial strains used contributed to the high rates of intestinal ischemia, necrosis, and mortality in the treated group. However, their meta-analysis of 13 randomized controlled trials (RCTs) found no statistically significant difference in mortality, infected pancreatic necrosis, and total infections between the pro-, pre-, and symbiotic-treated groups and the control group. On the other hand, a reduction in the length of the hospital stay was observed in the treated group [34]. You-Dong W. et al. enrolled 128 patients in a randomized, double-blind, placebo-controlled trial and divided them into two groups: the placebo group and the group treated with probiotic capsules of Bacillus subtilis and Enterococcus faecium [35]. Probiotics intake seems to be safe and effective in shortening the length of hospital stay in patients with mild acute pancreatitis, relieving abdominal pain, and reducing the time to oral food intake [35]. In addition, in critically ill patients (e.g., patients with acute pancreatitis in the intensive care unit), it has been demonstrated that altering the composition of the microbiota can lead to dysbiosis, which contributes to disease progression. Probiotics, prebiotics, and symbiotics may be useful in promoting the restoration of the intestinal epithelial barrier, preventing adhesion and colonization, and implementing the immune system [36]. The role of fecal microbiota transplantation (FMT) has not been adequately studied in the context of acute pancreatitis, but its potential effect in restoring intestinal microbiota homeostasis may represent an interesting future option for critically ill patients with a disrupted intestinal barrier. In a case report, FMT was described as an effective and safe treatment for a patient with acute pancreatitis and severe Clostridium difficile infection [37].

## 4. Chronic Pancreatitis

Chronic pancreatitis (CP) is a fibro-inflammatory syndrome of the pancreas in individuals with genetic, environmental, and/or other risk factors who develop persistent pathologic responses to parenchymal injury or stress. Persistent inflammatory episodes lead to fibrotic tissue replacement, resulting in exocrine and endocrine pancreatic insufficiency varying degrees and structural changes such as increased density of the parenchyma, atrophy of the gland, calcification, pseudocysts, and irregularities of the main pancreatic duct and its lateral branches [38]. Several studies investigated the composition of the gut microbiota in patients with chronic pancreatitis and its alteration at different stages of the disease. Patients with chronic pancreatitis show a high prevalence of dysbiosis of the gut microbiota with decreased diversity and richness [39]. Compared to healthy controls, the gut microbiota of CP patients shows a decrease in Firmicutes and Actinobacteria and an increase in Proteobacteria filum [39]. Moreover, Eubacterium rectale group, Coprococcus, Sutterella, and Eubacterium ruminantium group are the dominant genera in the fecal microbiome of CP patients with exocrine pancreatic insufficiency (PEI), while Pseudomonas, Fusobacterium, and Ruminococcus gnavus group are the dominant genera in the fecal microbiome of CP patients without PEI. Bifidobacterium and Lachnoclostridium show a positive correlation with fecal elastase 1 [39]. Chunhua W. et al. examined the gut microbiota of the cerulein-induced mouse model CP and confirmed the decrease in bacterial richness and diversity, with lower levels of Firmicutes and higher levels of Bacteroidetes, Actinobacteria, and Verrucomicrobia [40]. Although the cerulein-induced CP mice group showed a significant decrease in body weight compared to the control group, no correlation was found between the changes in gut microbiota and the changes in body weight. Patients with CP also have an increase in facultative pathogenic organisms, such as Enterococcus, Streptococcus, and Escherichia-Shigella [41]. Members of Escherichia-Shigella are associated with the production of lipopolysaccharide (LPS), which stimulates the inflammatory cascade and activates Toll-like receptor 4 [42]. LPS may also modulate the TGF-β1 pathway, leading to the activation of pancreatic stellate cells and a consequent increase in collagen production, resulting in pancreatic fibrosis [43]. Pancreatic endocrine insufficiency (diabetes type 3c) is another clinical consequence of CP. Pancreatic endocrine insufficiency is determined by the loss of pancreatic tissue due to chronic fibrosis, resulting not only in a deficiency of insulin, but also of counter-regulatory hormones (such as glucagon) [44]. Type 3c diabetes is a complex disease associated with large fluctuations in blood glucose levels that are difficult to control [45]. The alteration of the gut microbiota associated with CP may contribute to the metabolic abnormalities associated with the disease, and in particular to the onset of diabetes. Jandhyala S.M. et al. studied the changes in the gut microbiota of 30 patients with CP (16 without and 14 with diabetes) and 10 healthy controls [17]. They reported a decrease in the abundance of Faecalibacterium prausnitzii in patients with CP, and especially in those with type 3c diabetes. The abundance of Faecalibacterium prausnitzii showed a negative correlation with fasting blood glucose and postprandial blood glucose, while a positive correlation was found with plasma insulin levels. Patients with type 3c diabetes and PEI had lower levels of Bifidobacterium compared to patients without PEI. Pancreatic enzyme replacement therapy (PERT) is often used in patients with CP and PEI-related symptoms. Two studies investigated the change in gut microbiota composition in animal models after supplementation with PERT. Nishiyama et al. examined the gut microbiota in the feces of mice treated with PERT compared to controls [45]. They conclude that PERT improves PEI-associated symptoms by restoring digestive activity, promoting fat absorption, and altering the gut microbiota of mice. According to PERT, there is evidence of an increased relative abundance of Akkermansia muciniphila (belonging to the Verrucomicrobia phylum), bacteria capable of normalizing the intestinal barrier and reducing intestinal barrier disruption, and Lactobacillus Reuteri, capable of maintaining intestinal epithelial regeneration and homeostasis, in treated mice [46,47]. The second study conducted by Ritz et al. concluded that dysbiosis could be normalized after PERT [48]. In their pig model of exocrine pancreatic insufficiency, a decrease in intestinal pathobionts such as Escherichia-Shigella, Acinetobacter, or Stenotrophomonas was observed after PERT. External factors such as smoking and alcohol consumption are known causes of CP, and can simultaneously affect the composition of the gut microbiota. Interestingly, children with genetic CP, without external predisposing factors, also show alterations in the gut microbiota. Wei W. et al. studied the gut microbiota of children with genetic CP compared to healthy controls and non-genetic CP [49]. They documented a decreased abundance of Butyricococcus in CP with CFTR mutations, in combination with mutations in CASR, CTSB, SPINK1, and/or PRSS1 and an increased abundance of Ruminococcaceae in CP with mutations in CASR, CTSB, SPINK1, and/or PRSS1. Conversely, CP without gene mutations showed an increased abundance of Veillonella and a decreased abundance of Phascolarctobacterium. This study highlights that those specific functional genes involved in the occurrence of pancreatic diseases may also determine specific changes in the gut microbiota. Quadros dos Santos P. et al. conducted a prospective, randomized, controlled, double-blind intervention study to determine whether the use of symbiotics can alter the natural history of chronic pancreatitis [50]. The treatment group received a symbiotic consisting of Lactobacillus casei, Lactobacillus rhamnosus, Lactobacillus acidophilus, Bifidobacterium bifidum, and fructooligosaccharides, while the control group received a medium-absorbing complex carbohydrate. The treatment group experienced a significant decrease in bowel frequency and improvement in laboratory tests (increase in hemoglobin, hematocrit, red blood cells, total lymphocyte count, serum magnesium, albumin, and a decrease in serum total cholesterol). The potential role of symbiotic treatment in altering the nutritional status of CP patients represents a novel and interesting approach to the treatment of CP, but further studies in this area are needed to make clear recommendations. In addition, restoring the balance of the gut microbiota through FMT could theoretically be a useful treatment for managing patients with PEI who are resistant to standard treatment. However, there are currently no studies investigating the role of FMT in CP.

## 5. Autoimmune Pancreatitis

Autoimmune pancreatitis (AIP) is a chronic fibro-inflammatory disease of the pancreas, with an autoimmune etiology that often begins with obstructive jaundice, with or without pancreatic mass [51]. Two different types of AIP are described:

(1) Type 1 is an IgG4-associated pancreatitis and can be considered a manifestation of systemic immunoglobulin (Ig)G4-associated disease (IgG4-RD) characterized by elevated serum IgG4 levels, extrapancreatic lesions, and infiltration of the gland with IgG4 plasma cells and lymphocytes, and histologically referred to as lymphoplasmacytic sclerosing pancreatitis (LPSP);
(2) Type 2 is characterized by a younger age at presentation, absence of extrapancreatic involvement, a histopathological pattern of chronic pancreatitis referred to as idiopathic ductocentric pancreatitis with intraluminal and intraepithelial neutrophils in the pancreatic ducts and acini, with no or very few IgG4-positive plasma cells without systemic involvement [51].

Several studies demonstrate that innate immunity plays an important role in the development of this disease. For example, the massive infiltration of TLR-7 positive M2 macrophages in pancreatic tissue in patients with AIP has been described [52]. Another study shows how the activation of interferon regulatory factor 7 (IRF 7) was induced in pancreatic plasmacytoid dendritic cells (pDCs) in experimental AIP in mice, and that siRNA-mediated suppression of IRF7 expression prevented the development of AIP, leading to a reduction in the accumulation of pDCs in the pancreas and the production of IFN-α and IL-33 [53]. Other evidence comes from the detection of pDCs expressing IL-33 and the accumulation of IFN-α in the human pancreas with IgG4-released AIP, but not in human tissues with chronic alcoholic pancreatitis [54]. Due to the strong correlation between the microbiota, innate immunological response, and autoimmune disease development, the notion that the microbiota may play a crucial role in the pathogenesis of AIP is growing, and more and more studies are focusing on the analysis of the microbiota in patients with such pathology [55]. Broad-spectrum antibiotics have been shown to prevent the development of induced AIP in mice. Sterilization of the gut resulted in a significant decrease in the accumulation of pDC in the pancreas producing IFN-α and IL-33 [56]. In addition, the transfer of gut microflora from mice with a severe experimentally induced form of AIP promotes the accumulation of IFN-α and IL-33 producing pDCs in the pancreas. Alterations were also noted in the composition of the fecal microbiota in AIP-induced mice. Experimental AIP was induced with an intraperitoneal injection of polyinosinic polycytidylic acid (poli (I: C)). Bifidobacterium was more abundant in mice treated with poli (I: C). This may lead us to believe that dysbiosis may play a key role in the development of AIP. In contrast, other studies suggest that the presence of Bifidobacterium in the intestines of mice may be a consequence of the type I IFN response evident in AIP, rather than the cause [53,57]. Therefore, it cannot be excluded that Bifidobacterium plays a pathogenic role in AIP. One of the recent studies shows that Klebsiella pneumonia and microbe-associated molecular patterns (MAMPs) can activate pDCs and M2 macrophages in the pancreas to produce IFN-α and IL-33 [56]. The accumulation of pDCs and M2 macrophages in the pancreas leads to the infiltration of immune cells, including IgG4-expressing plasmocytes, and fibrosis. Kamata K. et al. studied how the composition of the fecal microbiota changes after treatment with prednisolone. Under corticosteroid, there is evidence of the complete disappearance of Klebsiella spp. and reduction of Fusobacterium spp. from the intestines of AIP patients, while the relative abundance of Fuminococcus app. increased [56]. The above results led them to suggest the role of pathogenicity of K. Pneumoniae in AIP. Oral administration of heat-killed K. pneumonia in combination with an injection of polyinosinic polycytidylic acid showed that the severity of experimental AIP was greater in mice that received both heat-killed K. pneumonia and an injection of poly(I: C) than in mice given only one agent. The accumulation of pDCs in the pancreas was also higher in mice that received both treatments [58]. We can therefore conclude that dysbiosis may play a significant role in enhancing AIP, or even in the development of AIP and IgG4-RD. Manipulation of the gut microbiota through prebiotics, probiotics, symbiotics, and FMT could be a new approach, not only for treatment but, more importantly, for preventing the onset and slowing the progression of AIP. RCTs are needed to evaluate their role in daily clinical practice.

## 6. Pancreatic Cancer

Pancreatic cancer is the seventh leading cause of cancer-related death worldwide, in both men and women, and the tumor with the highest incidence-mortality ratio [59]. It has been predicted that pancreatic ductal adenocarcinoma (PDAC) will be the second leading cause of death within the next 10 years [60]. The incidence of pancreatic cancer varies between countries, with the highest incidence in North America and Western Europe and the lowest incidence in Central Africa and South-Central Asia. Pancreatic cancer today is still associated with a poor prognosis, even when initially resectable. PDAC is often diagnosed at advanced stages and is associated with a very poor five-year survival rate of 2–9% [61]. The poor outcomes are largely due to the lack of early clinical symptoms, the likelihood of subclinical metastatic disease at presentation, the late stage at presentation, the lack of early and reliable diagnostic biomarkers, and the complex biology surrounding the extensive desmoplastic microenvironment of the pancreatic tumor [62]. Many studies analyzed the role of microbiota in patients with PDAC and its possible involvement in pathogenesis, diagnosis, treatment, and prognosis.

### 6.1. Oral Microbiome in PDAC

It is now known that periodontal disease, including gingivitis and periodontitis, and tooth loss are considered independent risk factors for the development of pancreatic cancer [63,64]. Although the exact relationship between the oral microbiota and the pathogenesis of pancreatic cancer is unknown, the translocation of microbes via the bile ducts, pancreatic ducts, or bloodstream into the pancreatic parenchyma is considered a possible mechanism [62]. Among the bacterial species present in the oral cavity of patients with PDAC, an increase in Leptotrichia and Porhyromonas and a decrease in Neisseria and Aggregatibacter were found compared to healthy controls [65]. Fan X. et al. documented the association between oral cavity bacteria such as Porphyromonas gingivalis and Aggregatibacter actinomycetemcomitans with a higher risk of developing pancreatic cancer in a prospective study [66]. The reported oral cavity pathogens mainly involved in the development of pancreatic cancer are Porphyromonas gingivalis, Fusobacterium, Neisseria elongata, and Streptococcus mitis. Among these, P. gingivalis is the periodontal pathogen most commonly associated with PDAC [67]. A systematic review by Memba R. et al. showed that the presence of lower levels of Neisseria elongate and Streptococcus mitis and higher levels of Granulicatella adiacens in saliva samples are associated with an increased risk of pancreatic cancer [68]. Lu H. et al. analyzed the composition of the tongue coating microbiome in patients with adenocarcinoma of the pancreas compared to healthy controls, and found a significant increase in the diversity of the microbiome in patients with pancreatic cancer [67]. Fusobacteria represented the most abundant phylum in patients with pancreatic cancer, followed by Actinobacteria and Clostridia. The most significant differences between the two groups were the low levels of Haemophilus and Porphyromonas, and the high levels of Leptotrichia and Fusobacterium in patients with PDAC. [69]. In conclusion, oral microbiota seems to play a significant role as a non-invasive diagnostic and prognostic biomarker for PDAC.

### 6.2. Gut Microbiota and Intratumor Microbiota in PDAC

Several studies reported changes in the gut microbiota in patients with PDAC compared to healthy controls. Patients with PDAC have lower gut microbiota diversity and a unique microbial profile, with an increase in potential pathogens (e.g., Enterobacteriaceae, Veillonellaceae, Streptococcaceae) and LPS-producing bacteria (e.g., Prevotella, Hallella, and Enterobacter), and a decrease in some probiotics (i.e., Bifidobacterium) and butyrate-producing bacteria (i.e., Coprococcus, Clostridium IV, Blatuia, Flavonifractor, and Anaerostipes) [70]. Another study confirmed that alpha diversity was reduced in the duodenal fluid of patients with PDAC compared to age-matched controls with normal pancreas and with pancreatic cyst [71]. Higher levels of Bifidobacterium genera were also detected in the duodenal fluid of patients with PDAC. In addition, duodenal fluid from patients with short-term surviving PDAC was enriched in Fusobacteria and Rothia [71]. Del Castillo et al. documented that the gut microbiota and intratumoral microbiota of patients with PDAC differed from the gut microbiota and intrapancreatic microbiota of control subjects who had not died of cancer [72]. In the first group, there was a lower presence and relative abundance of Lactobacillus, while there was a significant increase in Porphyromonas, Capnocytophaga, Prevotella Selenomonas, and Fusobacterium [72]. The intestinal microbiota of patients with PDAC had a higher proportion of Proteobacteria, Actinobacteria, Fusobacteria, and Verrucomicrobia compared to healthy controls. Proteobacteria were also prominent in these patients and were associated with advanced disease stages [12]. Maekawa et al. found that the pancreatic juice of patients with pancreatic cancer or duodenal cancer/biliary duct cancer was enriched with several bacterial species, particularly Enterococcus and Enterobacter [73]. These findings triggered new studies to analyze the intratumoral microbiota and its correlation with carcinogenesis. A recent study showed that cancerous pancreases harbor a significantly richer microbiome compared to normal pancreases in mice and humans [12]. It also showed how dysbiosis plays a key role in oncogenesis and how antibiotic therapy reduces tumor progression, and protects against preinvasive and invasive pancreatic ductal adenocarcinomas in mice. Evidence suggests that the microbiota is a potent modulator of the tumor microenvironment and promotes tumor progression by inducing peritumoral immunosuppression. Evidence for this is the immunogenic reprogramming of mice with antibiotic ablation, leading to a decrease in myeloid-derived suppressor cells and an increase in M1 macrophage differentiation, which promotes CD8+T cell activation. The microbiota was also able to induce immunosuppression through the activation of TLR, and the immunosuppressive effects of the PDA microbiome were absent in macrophages that lacked TLR signaling. In contrast, Bifidobacterium Pseudolongum was found to play a role in pancreatic oncogenesis through a TLR-dependent pathway. Therefore, cell-free extracts from B. pseudolongum can polarize macrophages to upregulate tolerogenic cytokines such as IL-10. These data suggest that the intratumoral microbiota is capable of modulating immunosuppression, and may be a potential therapeutic target in modulating disease progression. Riquelme et al. showed that the composition of the tumoral microbiome influences the host immune response and natural disease progression by interacting with the immune system and promoting cancer-associated inflammation [74]. When comparing patients with resectable PDAC and analyzing survival rates, long-term survivors (LTS) (more than 5 years after surgery) had higher tumor alpha diversity than short-term survivors (STS). The intratumoral microbiota may contribute to the anti-tumor immune response and promote CD8+ T cell recruitment and activation. Indeed, a higher density of CD8 T+ cells was found in LTS tumor samples. A recent study has demonstrated the presence of intracellular bacteria in both tumor cells and immune system cells [75]. However, it is not yet clear whether their presence plays a pathogenetic role or merely reflects tumor colonization because of the leaky vasculature of the tumor, which allows the spread of bacteria in the context of tumor-induced immunosuppression. The microbiota in pancreatic cystic neoplasms (PCNs) was also analyzed to test whether bacteria might be involved in carcinogenesis. The most common PCNs are intraductal papillary mucinous neoplasms (IPMNs), which may follow the dysplasia-carcinoma sequence leading to malignant transformation [76]. It has been shown that intracystic bacterial DNA copy number was significantly higher in malignant IPMN (high-grade dysplasia or invasive cancer) compared to non-IPMN. IPMN with high-grade dysplasia also showed enrichment of Fusobacterium nucleatum and Granulicatella adiacens. Further studies are needed to understand the role of the gut and intracystic microbiota in the malignant transformation of IPMN [77].

Other studies focused on the role of microbiota as a prognostic tool to determine survival in patients with pancreatic cancer. Riquelme et al. demonstrated that LTS patients have an enrichment of 3 bacterial tumor taxa: Saccharopolyspora, Pseudoxanthomonas, and Streptomyces. These three taxa, as well as Bacillus clausii, which belongs to a different genus than the three described above, could be highly predictive of long-term survival in patients with pancreatic cancer [74]. The microbiota could also play a role as a potential biomarker in the differential diagnosis of pathologies that can mimic PDAC, such as autoimmune pancreatitis [78]. When analyzing the microbiota of patients with adenocarcinoma and autoimmune pancreatitis, Zhou et al. found a significant difference in bacterial types and fecal butyrate content [79]. In patients with PDAC, there was a large decrease in Firmicutes (mainly butyrate-producing bacteria), and an increase in Proteobacteria (mainly Gammaproteobacteria). Eubacterium rectale, Eubacterium ventrisum, and Odoribacter splanchnicus were among the most important biomarkers in distinguishing pancreatic adenocarcinoma from healthy controls and individuals with autoimmune pancreatitis. Geller et al. have shown that the presence of Gammaproteobacteria in pancreatic cancer samples can influence the response to gemcitabine, one of the most commonly used chemotherapeutic agents [80,81]. Indeed, Gammaproteobacteria are able to convert the chemotherapeutic agent gemcitabine (2′,2′-difluorodeoxycytidine) to its inactive form, 2′,2′-difluorodeoxyuridine. Moreover, in this study, the presence of bacteria was detected in 76% of tumorous pancreas samples compared to 15% of normal pancreas samples, with Gammaproteobacteria being the most frequently isolated bacteria [82]. In addition, the presence of intratumoral LPS detected by immunohistochemistry was present in approximately 24.5% of pancreatic adenocarcinoma samples [83]. Moreover, the overall survival of LPS-positive patients was lower compared to LPS-negative patients. LPS is considered a marker of colonization with Gram-negative bacteria, and may be a negative predictor of gemcitabine efficacy in advanced-stage pancreatic cancer [83]. Further studies are needed to validate the possibility of combining chemotherapy and adjuvant antibiotic treatment in LPS-positive tumors, in the hope of increasing the unfortunately still suboptimal treatment efficacy. Another study documented the presence of intratumoral LPS in most human pancreatic cancer tissues (51/62). In addition, LPS has been shown to induce intratumoral lymphocyte infiltration and PD-L1 expression via the TLR4 pathway, which may promote tumor immune escape [84]. These data suggest that circulating LPS may be considered a predictive factor for response to PD-1/PD-L1 immune checkpoint treatment, and may play a potential role in enhancing PD-1/PD-L1 ICB efficacy as an immunological adjuvant [84]. The role of probiotics has been investigated as adjuvant therapy to control the progression of pancreatic cancer. In a mouse model of PDAC, combination therapy with gemcitabine and probiotics (Lactobacillus paracasei GMNL-133 and Lactobacillus reuteri GMNL-89) was associated with lower levels of PanIN formation. Moreover, the intake of high-dose probiotics alone also had an inhibitory effect on the occurrence of PanIN. These data suggest that probiotics may increase the efficacy and tolerability of standard chemotherapy in cancer patients [85]. Interestingly, a diet enriched with prebiotics can modulate the expression of miRNA target genes involved in tumor growth, cell migration, and metastasis occurrence [86]. These data suggest that gut dysbiosis may influence tumorigenesis and cancer progression. Restoring the balance of the gut microbiota with FMT could be a useful tool in the treatment of PDAC [87]. Probiotics, prebiotics, symbiotics, and FMT may also play a crucial role in the treatment of cancer cachexia, one of the most common clinical manifestations of PDAC [88].

## 7. Non-Alcoholic Fatty Pancreatic Disease (NAFPD)

NAFPD is a long-standing disease that has attracted considerable interest in gastroenterology in recent years [89]. The accumulation of fat in the pancreatic parenchyma has been referred to by various terms over the years (fatty pancreas, pancreatic steatosis). However, these definitions have features that distinguish them from each other. Generally, the term pancreatic stratosis is used in the case of simple infiltration of pancreatic tissue with fat cells (e.g., in obese individuals). In contrast, NAFPD is used to identify the accumulation of liver fat associated with metabolic syndrome and obesity [90]. NAFPD has particular pathophysiological implications: It is associated with beta-cell dysfunction and impaired carbohydrate metabolism [89]. NAFPD is also associated with the development of non-alcoholic steato-pancreatitis (NAPS) and pancreatic cancer [91,92,93]. NAFPD and nonalcoholic fatty liver disease (NAFLD) have been linked to metabolic syndrome, and they likely share a similar pathogenesis [94,95]. The gut microbiota may trigger NAFLD through several mechanisms: Increase in intestinal permeability, modulation of the innate immune system, alteration of bile acid turnover, increase in endogenous ethanol production, and triggering of direct and indirect inflammatory stimuli [96]. Similar mechanisms could be claimed for the pathogenesis of NAFPD. Although there are no recent studies investigating the possible role of microbiota in pathogenesis and thus of probiotics in the prevention and treatment of NAFPD, the strong association between microbiota, metabolic syndrome and NAFLD opens a crucial line for future research evaluating the role of gut microflora in NAFPD [97,98].

## 8. Discussion and Conclusions

The role of the gut microbiota in pancreatic physiology and pathology has been extensively studied. The tight junctions of the pancreatic ductal epithelium and antimicrobial peptide secretion provide a barrier to bacterial translocation. However, alteration of the balance between the gut microbiota and the pancreatic barriers may lead to the opportunistic colonization of the pancreatic parenchyma [99]. Furthermore, the pancreas could be affected not only by direct bacterial colonization, but also by an indirect effect of small molecules and toxins derived from disturbed gut microbiota [100]. Inflammatory pancreatic diseases (acute pancreatitis, chronic pancreatitis, and autoimmune pancreatitis) continue to be associated with high mortality and morbidity. Pancreatitis, both acute and chronic, is not only a local disease, but determines a systemic inflammatory and immune response. There is undoubtedly a close relationship between the gut microbiome, dysbiosis, and the progression of acute pancreatitis (Table 1). Several studies have shown that severe AP is associated with an increase in the abundance of Enterobacteriaceae, Enterococcus, Bacteroides, Escherichia-Shigella, and a decrease in Bifidobacterium [25]. Therefore, a positive correlation between disturbed gut microbiota and systemic inflammation has been described in severe AP [24]. Although the association between dysbiosis and the severity of AP has been widely confirmed, the use of the gut microbiota as a therapeutic target is still debated. Some studies have been conducted on the use of prebiotics, probiotics, or symbiotics, but with conflicting results [32,33]. The role of the gut microbiota in chronic pancreatitis has been studied in terms of etiology, symptomatology, and treatment. The impairment of the gut microbiota is a common finding in chronic pancreatitis with a decrease in Firmicutes and Actinobacteria and an increase in Proteobacteria filum [38]. Interestingly, different alterations in gut microbiota have been described in different types of genetic chronic pancreatitis, suggesting a specific interaction between pancreas and gut microbiota [48]. Moreover, the role of dysbiosis has been described in patients with PEI, and especially in those who do not respond to PERT [44]. From this point of view, dysbiosis could be considered as a therapeutic target, and prebiotics, probiotics, and symbiotics could become an available treatment for patients with chronic pancreatitis who do not respond to standard treatments [49]. Changes in gut microbiota have been described also in patients with cystic fibrosis and pancreatic involvement. Patients with cystic fibrosis and pancreatic insufficiency showed lower alpha diversity of the gut microbiota compared to healthy controls and patients with normal pancreatic exocrine function [101]. A possible role of the gut microbiota in the pathogenesis of AIP has also been postulated, with a suspected link between gut microbiota, immune system, and pancreas [55]. The profile of the gut microbiota appears to differ between chronic and autoimmune pancreatitis. The association of each pancreatic pathology with its specific gut microbiota could be a potential biomarker, and be used as a diagnostic tool.

The study of the microbiota in pancreatic adenocarcinoma has shown that it may play a role in early diagnosis, pathogenesis, prognosis, and therapy (Table 2). The Cancer Genome Atlas (TCGA) characterized the cancer microbiome of 33 different cancers, and found unique microbial signatures in tissues and blood of the major cancers [102]. Microbiota alterations or specific cancer microbiota can be used as early biomarkers that can have a strong impact on the long-term survival of this disease with a poor prognosis. The microbiota has emerged as a potential non-invasive diagnostic tool, which is one of the most important challenges for microbiota research [68]. The analysis of microbiota has helped us to better understand the underlying mechanisms of pancreatic cancer pathogenesis. It has been demonstrated that the microbiota can modulate the immune system and induce peritumoral immunosuppression [12]. The microbiota could also be a valid biomarker for predicting PDAC risk and prognosis. The diversity of the tumor microbiome has a strong impact on the survival of PDAC patients [74]. Moreover, alpha diversity may be a predictor of survival in patients with resected pancreatic cancer [71].

Finally, the microbiota is involved in the metabolism of chemotherapeutic agents, which could influence the response to therapy. The microbiota is also able to modulate the tumor microenvironment, which could influence the efficacy of immunotherapy in pancreatic cancer.

In conclusion, the manipulation of the gut microbiota may be a promising treatment in the management of pancreatic diseases. Prebiotics, probiotics, symbiotics, and FMT are now widely used in daily practice for the treatment of various gastroenterological and non-gastroenterological diseases. Many studies have demonstrated the association of gut microbiota and dysbiosis with pancreatic inflammation and oncological diseases. Indeed, the gut microbiota may play a role in the pathogenesis, progression, and treatment of pancreatic diseases. Although our knowledge of the composition of the gut microbiota and its alterations in pancreatic diseases has increased in recent decades, little is known about how to translate in clinical practice this information to prevent and treat pancreatic diseases. Randomized controlled trials are needed to evaluate the role of these therapeutic options (prebiotics, probiotics, symbiotics, and FMT) in the treatment and prevention of pancreatic diseases.

## Figures and Tables

**Table 1:** Microbiota changes in inflammatory pancreatic diseases: acute pancreatitis, chronic pancreatitis, and autoimmune pancreatitis. ↑: increased levels; ↓: reduced levels.

Author, Year	Study Population	Material	Disease	Microbial Changes
C. Tan, 2015 [20]	Humans	Fecal and blood samples	Severe acute pancreatitis	↑ Enterococcus ↓ Bifidobacterium and increased IL-6 levels
X. M. Zhang, 2018 [21]	Humans	Fecal samples	Acute pancreatitis	↑ Bacteroidetes and Proteobacteria ↓ Firmicutes and Actinobacteria
S. Yu, 2020 [22]	Humans	Fecal samples	Acute pancreatitis	↑ Bacteoides in mild AP ↑ Escherichia-Shigella in mild-severe AP, ↑ Enterococcus in severe AP
Q. Li, 2013 [23]	Humans	Rectal swab and peripheral blood samples	Acute pancreatitis	↑ Escherichia-Shigella
Y. Zhu, 2019 [24]	Humans	Fecal samples	Acute pancreatitis	↓ Blautia and Bifidobacterium
X. Hu, 2021 [17]	Humans	Fecal samples	Hypertriglyceridemia-induced pancreatitis	↓ Lachnospiraceae and Bacteroidaceae
C.H. Zhou, 2020 [35]	Humans	Fecal samples	Chronic pancreatitis	↑ Proteobacteria ↓ Firmicutes and Actinobacteria
C.H. Zhou, 2020 [35]	Humans	Fecal samples	Chronic pancreatitis with pancreatic exocrine insufficiency	↑ Coprococcus, Sutterella, Eubacterium-ruminantium
C.H. Zhou, 2020 [35]	Humans	Fecal samples	Chronic pancreatitis without pancreatic exocrine insufficiency	↑ Pseudonomas, Fusobacterium and Ruminococcus-gnavus
C. Wu, 2021 [36]	Mice	Fecal samples	Experimental induced chronic pancreatitis	↑ Bacteroidetes, Actinobacteria and Verrucomicrobia, ↓ Firmicutes
F. Frost, 2020 [37]	Humans	Fecal samples	Chronic pancreatitis	↑ Enterococcus, Streptococcus and Escherichia-Shigella
S.M Jandhyala, 2017 [15]	Humans	Fecal samples	Chronic pancreatitis and 3c diabetes	↓ Faecalibacterium prausnitzii and Bifidobacterium
H. Nishiyama, 2018 [41]	Mice	Fecal samples	Chronic pancreatitis after PERT	↑ Akkermansia muciniphila and Lactobacillus reuteri
S. Ritz, 2020 [44]	Porcine	Fecal samples	Chronic pancreatitis after PERT	↓ Escherichia-Shigella, Acinetobacter and Stenotrophomonas
W. Wang, 2020 [45]	Humans	Fecal samples	Genetic chronic pancreatitis	↑ Ruminococcaceae ↓ Butyricicoccus
W. Wang, 2020 [45]	Humans	Fecal samples	Chronic pancreatitis without gene mutations	↑ Veillonella ↓ Phascolarctobacterium
K. Kamata, 2019 [52]	Mice	Fecal samples	Experimental induced autoimmune pancreatitis	↑ Bifidobacterium
K. Kamata, 2020 [54]	Humans	Fecal samples	Autoimmune pancreatitis after treatment with prednisolone	↑ Fuminococcus ↓ Klebsiella and Fusobacterium

**Table 2:** Microbiota changes in pancreatic cancer. ↑: increased levels; ↓: reduced levels.

Author, Year	Study Population	Material	Disease	Microbial Changes
P.J. Torres, 2015 [62]	Humans	Saliva samples	Pancreatic adenocarcinoma	↑ Leptotrichia and Porhyromonas ↓ Neisseria and Aggregatibacter.
R. Memba, 2017 [65]	Humans	Saliva samples	Pancreatic adenocarcinoma	↑ Granulicatella adiacens ↓ Neisseria elongate and Streptococcus mitis
H. Lu, 2019 [66]	Humans	Tongue coat samples	Pancreatic adenocarcinoma	↑ Leptotrichia and Fusobacterium ↓ Haemophilus and Porphyromonas
Z. Ren, 2017 [67]	Humans	Fecal samples	Pancreatic adenocarcinoma	↑ Enterobacteriaceae, Veillonellaceae, Streptococcaceae, Prevotella, Hallella, Enterobacter ↓Bifidobacterium, butyrate-producing bacteria
S. Kohi, 2020 [69]	Humans	Duodenal fluid	Pancreatic adenocarcinoma	↑ Bifidobacterium
S. Kohi, 2020 [69]	Humans	Duodenal fluid	Short-term survival pancreatic adenocarcinoma	↑ Fusobacteria and Rothia
E. Del Castillo, 2019 [72]	Humans	Fecal samples	Pancreatic adenocarcinoma	↑ Porphyromonas, Capnocytophaga, Prevotella Selenomonas and Fusobacterium ↓ Lactobacillus
S. Pushalkar, 2018 [10]	Humans	Fecal samples	Pancreatic adenocarcinoma	↑ Proteobacteria, Actinobacteria, Fusobacteria, and Verrucomicrobia
T. Maekawa, 2018 [73]	Humans	Bile samples	Pancreatic adenocarcinoma	↑ Enterococcus and Enterobacter
S. Pushalkar, 2018 [10]	Mice and Humans	Fecal and pancreatic tumor samples	Pancreatic adenocarcinoma	↑ Bifidobacterium pseudolongum
R.A. Gaiser, 2019 [77]	Humans	Cyst fluid	IPMN with high-grade displasia and IPMN with cancer	↑ Fusobacterium nucleatum and Granulicatella adiacens
E. Riquelme, 2019 [74]	Humans	Pancreatic tumor samples	Long-term survival pancreatic adenocarcinoma	↑ Saccharopolyspora, Pseudoxanthomonas and Streptomyces
W. Zhou, 2021 [79]	Humans	Pancreatic tumor samples	Pancreatic adenocarcinoma	↑ Proteobacteria ↓ Firmicutes

## Data Availability

Not applicable.
